# Characterization of Antennal Sensilla and Immunolocalization of Odorant-Binding Proteins on Spotted Alfalfa Aphid, *Therioaphis trifolii* (Monell)

**DOI:** 10.3389/fphys.2020.606575

**Published:** 2020-12-17

**Authors:** Limei Song, Xuemin Wang, Yanqi Liu, Yinpeng Sun, Liping Ban

**Affiliations:** ^1^College of Grassland Science and Technology, China Agricultural University, Beijing, China; ^2^Institute of Animal Sciences, Chinese Academy of Agricultural Sciences, Beijing, China

**Keywords:** chemosensilla, immunocytochemistry, odorant-binding protein, *Therioaphis trifolii*, ultrastructure

## Abstract

The spotted alfalfa aphid [*Therioaphis trifolii* (Monell), Homoptera, Drepanosiphidae] is a well-known destructive pest that can significantly reduce alfalfa yields. Herein, the morphology of antennal sensilla of *T. trifolii* has been examined by using scanning electron microscopy and the ultrastructure of sensilla stellate and placoidea was described by transmission electron microscopy. Stellate sensilla, placoid sensilla, and coeloconic sensilla were found on the 6th segment, and a single sensillum placoidea was located on the 5th segment. Placoid sensilla were also present on the 3rd antennal segment of alate and apterous aphids, and the number was similar between two morphs. Two types of trichoid sensilla and coeloconic sensilla were found on the antennae, respectively. The results of ultrastructure showed that stellate sensilla are innervated by three neurons, while placoid sensilla present three groups of neurons, equipped with 2–3 dendrites in each neuron group. Immunocytochemical localization of odorant-binding proteins (OBPs) was performed on ultrathin sections of sensilla stellate and placoidea, and we observed that the antiserum against OBP6 intensively labeled all placoid sensilla from both primary and secondary rhinaria. OBP7 and OBP8 could also be detected in placoid sensilla, but less strongly than OBP6. In addition, OBP6, OBP7, and OBP8 were densely labeled in stellate sensilla, suggesting OBP6, OBP7, and OBP8 may sense alarm pheromone germacrene A in *T. trifolii*.

## Introduction

The spotted alfalfa/clover aphid, *Therioaphis trifolii* (Monell) (Homoptera: Aphididae), is a cosmopolitan pest of legumes, mainly in the tribes Trifoliae and Loteae (Blackman and Eastop, 2000). Spotted alfalfa aphid (SAA) damages the plants directly by sucking the juices from the leaves and tender stems and indirectly by vectoring plant-pathogenic viruses, severely interfering plant growth and affecting the quality and quantity of herbage produced (He and Zhang, 2006). Losses of alfalfa have been large, and the need for control of the insect has become of great economic importance. According to the reports, SAA severely inhibits seedling establishment and plant growth, affecting the quality and quantity of herbage produced, particularly hay, with an estimated 25% loss in production (He and Zhang, 2006).

Using insecticides to control aphid populations has become more difficulty since aphids develop insecticide resistance (Tang et al., 2017; Zhang et al., 2017a; Hanson and Koch, 2018; Koch et al., 2018). Thus, there is considerable interest in developing eco-friendly pest-control methods, with the use of semiochemicals as a distinct possibility. Searching for environmentally safe prevention and control strategies is extremely important. The behaviors of insects, such as locating food sources, mating partners, oviposition sites, choice of suitable hosts, and identifying predators, are frequently modulated or evoked by semiochemicals emitted by host plants or conspecifics (Zwiebel and Takken, 2004; Yoshizawa et al., 2011). The olfaction system plays a critical role in perceiving the semichemicals of insects (Karg and Suckling, 1999; Field et al., 2000). Aphids, like other insects, use semichemicals to direct much of their behaviors (Fan et al., 2015; Zhang et al., 2017b; Song et al., 2018).

It is well known that the antenna is one of the primary organs that insects use to recognize semiochemicals and environmental odors. In aphids, the antennal olfactory sensilla have been divided into primary rhinaria, second rhinaria, and trichoid sensilla according to external morphology (Shambaugh et al., 1978; Zhang and Zhang, 2000). The primary rhinaria occur on the 5th and 6th segments of the antenna and include several sensillum types. The second rhinaria, which are sensilla placoidea, in fact are located between the 3rd and 5th segments. The literature reported that two different types of trichoid sensilla have been identified based on the morphology (Bromley et al., 1980). The type I hair are found along the whole length of the antenna as far as the 6th segment primary rhinarium, while type II hair occur along the processus terminalis and on the tip of the antenna.

Aphids produce repellent droplets from the cornicles to alert nearby conspecifics to escape by walking away and dropping off the host plant to protect native populations from natural enemies or other dangers. These secretions contain alarm pheromone (Dixon, 1958; Dahl, 1971; Kislow and Edwards, 1972; Nault et al., 1973; Goff and Nault, 1974). The alarm pheromone plays an essential role in aphid’s behavior and has been applied to explore potential strategies for aphid population control (Li et al., 2017). Two primary alarm pheromones, (*E*)-β-farnesene and germacrene A, have been identified in aphids until now. (*E*)-β-Farnesene has been found in all studied species of subfamilies Aphidinae and Chaitophorinae, while germacrene A was identified only within the genus *Therioaphis* of the subfamily Drepanosiphinae (Bowers et al., 1972, 1977; Nault and Bowers, 1974; Nishino et al., 1977). Early study showed that aphids of genus *Therioaphis*, such as *T. trifolii*, are lack of response to (*E*)-β-farnesene (Nishino et al., 1977). Although biochemical research on the olfactory system of aphids is rapidly progressing, information about Drepanosiphinae’s aphids is still scanty and fragmentary. In insects, semiochemicals and environmental odors enter the sensillum lymph via pores in the cuticle of the sensilla and are carried by odorant-binding proteins (OBPs), transported through the sensillum lymph and finally reached sensory dendrites, where they activate membrane-bound odorant receptors (ORs) (Brito et al., 2016). OBPs, usually 14–20 kDa, are abundantly expressed in the lymph of chemosensilla and referred to as the solubilizer and carrier of hydrophobic pheromones and discrimination of semiochemicals (Qiao et al., 2009; Pelletier et al., 2010; Swarup et al., 2011; Sun et al., 2012). Early studies reported either or both of OBP3 and OBP7 might be involved in (*E*)-β-farnesene perception in most aphids, such as in *Rhopalosiphum padi* and *Acyrthosiphon pisum* (Qiao et al., 2009; Fan et al., 2017; Zhang et al., 2017a). Although previous study reported the alarm pheromone of *T. trifolii*, which OBP is involved in the perception of germacrene in *T. trifolii* are still unknown.

While biochemical research on the olfactory system of aphids is rapidly progressing, information at the anatomical level for SAAs *T. trifolii* (Homoptera, Drepanosiphidae) is still scanty and fragmentary. This study was conducted to investigate the function of antennae in *T. trifolii* by studying the distribution and fine structure of chemosensilla, using both scanning and transmission electron microscopy, and mapping the expression of OBPs in such sensilla. Our research offers data related to the candidate OBPs potentially involved in perception of semiochemicals in aphid *T. trifolii*, which will provide original strategies for aphid’s integrated management. Herein, we report on the morphology and ultrastructural characterization of the different types of antennal sensilla in SAA *T. trifolii* by scanning and transmission electron microscopy. In addition, the distribution and expression of OBP6, OBP7, and OBP8 in sensilla stellate and placoidea was investigated.

## Materials and Methods

### Insect Rearing

Spotted alfalfa aphid *T. trifolii* was reared on alfalfa (*Medicago sativa*) at 20–22°C, 60–70% relative humidity with a photoperiod of 16: 8 h (light: dark) at College of Grassland Science and Technology, China Agricultural University, Beijing, China.

### Scanning Electron Microscopy

For better confirmation of the number and types of sensilla on the antenna of *T. trifolii*, twenty alate and apterous adult aphids were used in this study for scanning electron microscopy (SEM). The heads of all samples were carefully excised with fine forceps under a stereomicroscope. The heads were first kept in 70% ethanol for 48 h at room temperature and then cleaned in an ultrasonic bath (250 W) for 5 s in the same solution. After dehydrated by an ethanol serial solution (30, 50, 70, 80, 90–100%) (Bock, 1987) in each case for 3 min, the dehydrated specimens were dried in Critical Point Dryer (LEICA CPD 030, Wetzlar, Germany) for 1.5 h. The dried head was mounted on holder and gold-sputtered in a Hitachi sputtering ion exchanger (HITACHI ID-5, Tokyo, Japan), and then the sensilla types were identified and counted in a HITACHI S-4800 SEM (Japan). Pictures were only adjusted for brightness and contrast.

### Transmission Electron Microscopy

The antennae used for Transmission electron microscopy (TEM) were excised and prefixed for 2 days with paraformaldehyde (4%) and glutaraldehyde (2.5%) in 0.1 M phosphate buffered saline (PBS, pH 7.2), then postfixed for 1 h with 1% OsO_4_ in 0.1 M PBS (pH 7.2), and followed by dehydration in an ethanol series solutions (30, 50, 70, 80, 90, 95–100%) for 3 min each. After being dehydrated with pure acetone three times for 10 min each, the samples were embedded in Epoxide resin 618 through mixtures of 2: 1, 1: 1, 1: 2 of acetone and Epoxide resin 618 (Serva, Heidelberg, Germany) and then kept in pure Epoxide resin 618 overnight. Polymerization was accomplished with heating from 30 to 60°C (5°C/6 h), at 60°C for 48 h in tightly closed gelatin capsules filled completely with the resin monomer. Ultrathin sections were cut with a diamond knife (Diatome, Bienne, Switzerland) on a Leica EM UC6 microtome (Wetzlar, Germany) and then mounted on Formvar-coated grids. The sections were observed on a HITACHI H-7500 TEM (Hitachi, Tokyo, Japan). Pictures were only adjusted for brightness and contrast.

### Immunocytochemistry

The antennae used were prefixed in paraformaldehyde (4%) and glutaraldehyde (2%) in 0.1 M PBS (pH 7.4) and then dehydrated in an ethanol series. The samples were embedded in LR White resin, and ultrathin sections were cut and mounted on Formvar-coated grids. For immunocytochemistry, the grids were subsequently floated, each time for 5 min, on 25-μL droplets of the following solutions, along with the procedure adapted from Steinbrecht et al. (1992). In brief, the grids with the sections were floated on solutions of PBG (PBS containing 50 mmol/L glycine) and PBGT (PBS containing 0.2% gelatin, 0.5% bovine serum albumin, and 0.02% Tween-20) twice for each solution, and then overnight at 4°C in primary antiserum (against OBP6, OBP7, and OBP8, respectively), or preimmune serum in PBGT. After six washings with PBGT, sections were incubated for 1 h with a secondary antibody in PBGT (1: 20) at room temperature, followed by two washings with PBGT, PBS glycine, and water. Each washing step was performed with 20-μL droplets for 5 min. Silver intensification (Danscher, 1981) was also applied to increase the size of the gold granules, followed by 2% uranyl acetate to increase the tissue contrast in TEM. Sections were then observed under a transmission electron microscope HITACHI H-7500 (Hitachi, Tokyo, Japan). Pictures were only adjusted for brightness and contrast.

In this study, the antisera against OBP6, OBP7, and OBP8 of *A. pisum* were kindly provided by Dr. Paolo Pelosi, University of Pisa (Qiao et al., 2009; Sun et al., 2012), and used as the primary antisera. The primary antiserum was used at dilutions 1: 1000. The serum from a healthy rabbit at the same dilution was used as the control. In the controls, the primary antiserum was replaced by a serum from a healthy rabbit at the same dilution. A secondary antibody was anti-rabbit IgG, coupled to 10-nm colloidal gold (AuroProbe^TM^ EM, GAR G10, Amersham). Image analysis was performed with ImageJ (developed at the United States National Institutes of Health).

## Results

**FIGURE 1:**
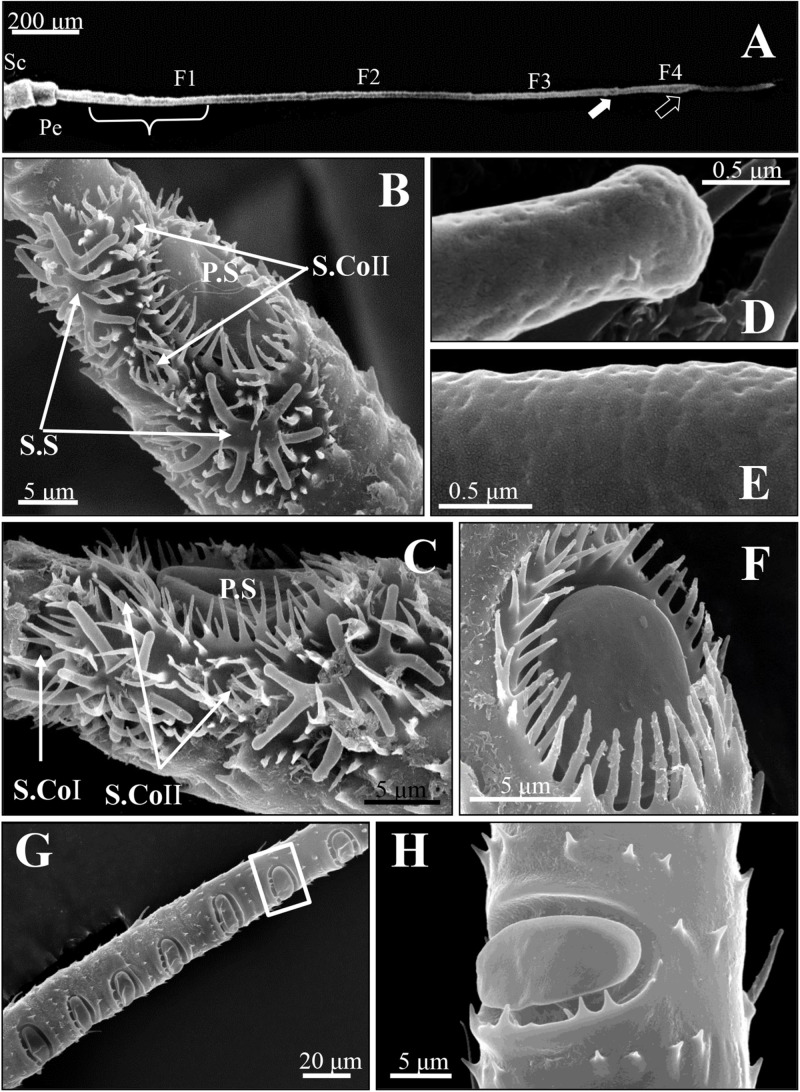
Scanning electron micrographs of antennal sensilla of spotted alfalfa aphid *Therioaphis trifolii* (Monell). **(A)** The antenna consists of six segments, including a scape (Sc), a pedicel (Pe), and four flagella (F1–F4). The primary rhinaria are located on the fifth (F3, white arrow) and sixth segments (F4, black arrow), the secondary rhinaria on the third segment (F1, brace). **(B,C)** The primary rhinaria on the 6th segment composed of one large placoid sensillum (P.S), two stellate sensilla (S.S), and two–three coeloconic pegs (S. CoI and S. CoII). **(D)** The stellate sensilla and **(E)** placoid sensilla at higher magnification, showing many pores on the surface of these sensilla. **(F)** The placoid sensillum (P.S) on the 5th segment of the primary rhinarium surrounded by a fringed cuticular ridge. **(G)** The distribution of the secondary rhinarium on the 3rd segment of the antennae. **(H)** The secondary rhinarium shown at high magnification, which was in the boxed areas in **(G)**.

The antennae of SAA *T. trifolii* are composed of three parts: a scape (Sc), a pedicel (Pe), and a long flagellum (F), with a total length of 1.8 mm (Figure 1A). The length of flagellum accounts for more than 80% of the whole the antenna and consists of four subunits, named F1–F4.

Four morphologically distinct types of sensilla were present on the entire surface of SAAs’ antennae, including placoid sensilla, stellate sensilla, coeloconic sensilla, and trichoid sensilla. The primary rhinaria of *T. trifolii* was found on the 5th and 6th segments of the antennae. The primary rhinarium on the 6th segment consists of one large placoid sensillum, two stellate sensilla, and two to three coeloconic sensilla (Figures 1B,C), with numerous pores penetrated the surface the former two types sensilla (Figures 1D,E). The primary rhinarium on the 5th segment is a single placoid sensillum similar to that on the 6th segment (Figure 1F). Numerous secondary rhinaria (placoid sensilla) are located on the 3rd segment (Figures 1G,H), and the numbers of placoid sensilla were similar between alate and apterous morphs, ranging from 5 to 12 (Table 1 and Supplementary Figure 1). There are two to three coeloconic sensilla in total, and they are classified into two types according to the terminal projections. Trichoid sensilla are classified into two types according to their morphology, which are along the whole length of the antenna. The number and the distribution of different sensilla on the antenna of *T. trifolii* are listed in Table 1 and Supplementary Figure 1. In addition, the expressions of OBPs in the antennal placoid and stellate sensilla of the aphid *T. trifolii* have been investigated by using immunocytochemical methods.

**TABLE 1:** The comparison of antennal sensilla abundance between spotted alfalfa aphid and other aphids.

**Aphid type**	**Primary rhinarium**			**Second rhinarium**		***S*. trichoidea (I/II)**	**References**
	***S.* placoid (large/small)**	***S*. stellate**	***S*. coeloconica (I/II)**	**Alate aphid**	**Apterous aphid**		
*Myzus persicae*	4 (2/2)	0	4 (2/2)	–	9∼10	24∼26 (20∼22/4)	Sun et al., 2013; Ban et al., 2015
*Acyrthosiphon pisum*	4 (2/2)	0	4 (2/2)	3∼5	14∼22	24∼26 (84∼95/3∼7)	Shambaugh et al., 1978; De Biasio et al., 2015
*Therioaphis trifolii*	2 (2/0)	2	2∼3 (0∼1/2)	6∼12	5∼10	33∼41 (29∼37/5)	This study
							

### Placoid Sensilla

The placoid sensilla of SAA are flat oval plates in a cavity. They constitute both the primary and secondary rhinaria of these aphids, but only the former are surrounded by a cuticular ridge (Figure 1F), whereas those of secondary rhinaria are only surrounded by a few small microtrichia at the proximal edge of the cavity (Figure 1H).

**FIGURE 2:**
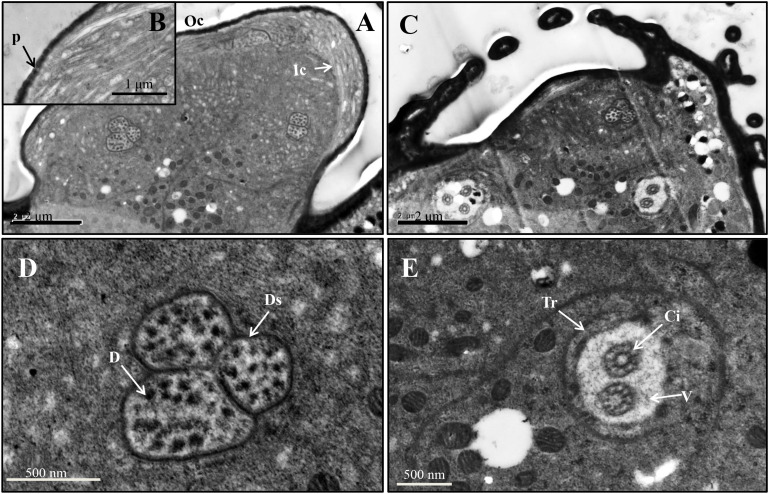
Ultrastructure of placoid sensilla on the 6th antennal segment of spotted alfalfa aphid. **(A)** The sensillum bears many pores on the surface. **(B)** Pores on the sensillum surface perforate the outer cuticle. **(C)** The dendrites of the sensillum are clustered into three groups, two of them containing three neurons while the other one only with two. **(D)** Each dendrite group was covered by dendritic sheath. **(E)** Each group of dendrites is subdivided into inner and outer segments by a short ciliary region. D, dendrite; Ds, dendritic sheath; V, vacuole; Tr, trichogen cell; P, pore; Oc, outer cuticle; Ic, inner cuticle; Ci, cilium.

For the primary rhinaria on the 5th segment and 6th segment, there is a single large placoid sensillum, respectively (Figures 1B,F). The large placoid sensillum on the 6th segment is approximately 12 μm in diameter and similar to the one on the 5th segment. Many pores are located on the surface of these sensilla (Figure 1E) and perforated the outer cuticle (Figures 2A,B). The dendrites of bipolar neurons within the placoid sensillum are clustered into three groups (Figure 2C), two of them containing three bipolar neurons (Figures 2C,D), while the third group has only two bipolar neurons (Figures 2C,E). The bipolar neurons are enclosed in a dendritic sheath (Figure 2D). The dendrite is subdivided into inner and outer segments by a short ciliary region, and each group of neurons is surrounded by trichogen cell (Figure 2E). Both the single placoid sensillum on the 5th segment and the secondary rhinaria along the third segment present internal structures similar to that of the placoid sensillum on the 6th segment.

### Stellate Sensilla

**FIGURE 3:**
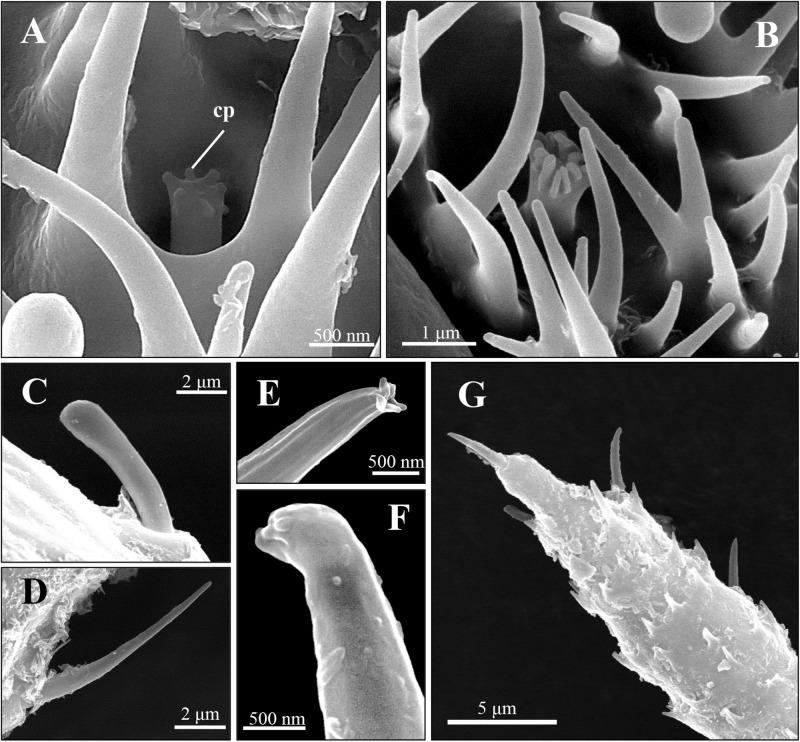
Scanning electron micrographs of coeloconic sensilla and trichoid sensilla of spotted alfalfa aphid antennae. **(A,B)** The coeloconic sensilla type I **(A)** and type II **(B)** on the 6th segment. **(C,D)** The trichoid sensilla type IA on antennal scape and pedicel **(C)** and trichoid sensilla type IB **(D)** are located from the 3rd segment to the 6th segment of antennae. **(E–G)** The trichoid sensilla type II **(E,F)** are distribution on the 6th segment tip **(G)**. Cp, cuticular projections.

Two stellate sensilla are present on the 6th antennal segment of *T. trifolii* as part of the primary rhinaria. These sensilla usually have six to eight branches and surrounded by a fringed cuticular ridge (Figure 1B). The branch is about 5 μm in length. Similar to placoid sensilla, many pores are present on the surface of the branches (Figures 1D, 3A) that penetrate the outer cuticle (Figure 3B). The diameters of the pores are about 30 nm (Figure 1D). The dendrites of bipolar neurons within these sensilla are clustered into one group, containing three bipolar neurons (Figure 3C). The dendrites embedded by dendrite sheath, which was disappeared at the distal ends of the dendrites. When the dendrites enter into the intercuticular space between the inner and outer cuticles, they are separated into dendritic branches and turn toward the distal end of the sensillum, occupying the whole space (Figure 3C). The space beneath the pore is filled with the sensillum lymph where the dendritic branches are located. The dendrite is also subdivided into inner and outer segments by a short ciliary region, surrounded by the trichogen cell (Figure 3D).

### Coeloconic Sensilla

**FIGURE 4:**
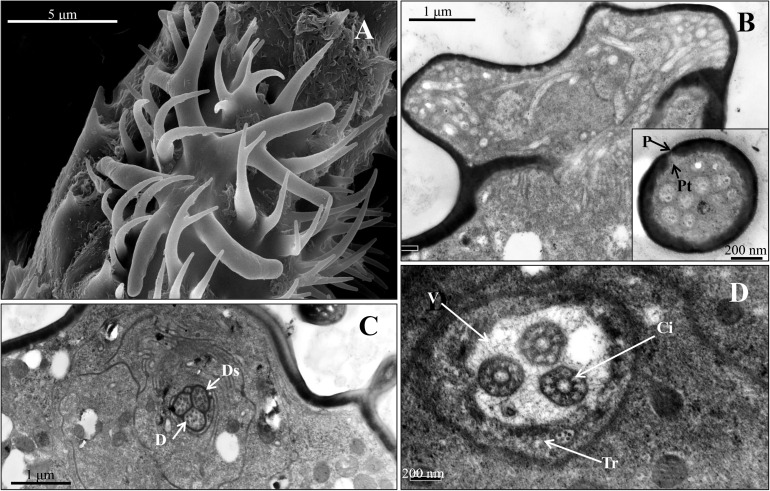
Stellate sensillum on the 6th antennal segment of spotted alfalfa aphid *T. trifolii*. **(A)** Scanning electron micrographs of stellate sensilla. **(B)** Transverse sections through the branch of the sensillum. **(C,D)** Transverse section showing three dendrites surrounded by the dendritic sheath **(C)**, and the ciliary regions of the sensillum **(D)**. D, dendrite; Ds, dendritic sheath; V, vacuole; Tr, trichogen cell; P, pore; Ci, cilium; Pt, pore tubules.

Two to three coeloconic sensilla are present on the 6th segment of the SAA antenna, and the distribution is shown as in Figures 1B,C. They are typical peg-in-pit sensilla, characterized externally by a round aperture (Figures 4A,B). The peg is 1.5 μm height and 0.6 μm diameter (Figures 4A,B). The peg terminates in many cuticular projections and exhibits a range of different shapes (Figures 4A,B). According to the terminal projections, the coeloconic sensilla were classified into two types. The cuticular projections of type I hair were usually characterized by a crown of six cuticular projections (Figure 4A), while type II hair exhibits a more complicated morphology of the peg ending with more cuticular projections, usually closely packed (Figure 4B). These sensilla of the SAA were similar to that found in peach aphids *Myzus persicae* (Ban et al., 2015).

### Trichoid Sensilla

Two distinct types of trichoid sensilla are present on the antenna of the SAA. The type I hair of trichoid sensilla is visible along the whole length of the antenna, and there is no pore on the surface and the tip of these trichoid sensilla (Figures 4C,D). Type II hairs were found only on the tip of antennae and uniporous (Figures 4E–F). Depending on external morphology, the type I is divided into two subtypes type IA (Figure 4C) and type IB (Figure 4D). The type IA hairs present a swollen tip with diameter 1.8 μm, and the base of the hair forms an oval-shaped plate, inserted into a ring-shaped socket. Type IA hairs are approximately 7.5 μm long and 1.2 μm wide of the base (Figure 4C). Type IB hairs are similar to type IA except for that they are equipped with a sharp tip with diameter 0.4 μm (Figure 4D). The type IA hairs present on the scape and pedicel, while type IB hairs are found from the 3rd segment to the 6th segment. Type II hairs present on the tip of the antenna, crowned by five of this sensilla (Figure 4G). Type II hairs are approximately 4–6 μm in length and 0.7–1.2 μm in width, inserted into a ring-shaped socket. A pore is found on the tip of type II hairs, while there are no pores on the surface of the sensilla (Figures 4E–G).

### Immunolabeling of OBPs

Immunocytochemical experiments were performed to investigate the cellular localization of OBP6, OBP7, and OBP8 in the SAA antennae. The results indicated that sensilla placoidea and stellate are labeled by OBP antisera except for sensilla trichodea and coeloconica. The distribution of OBPs and the labeling density in different sensilla placoidea and stellate are summarized in Table 2.

**TABLE 2:** Immunocytochemical localization of OBPs in placoid sensilla on the antennae of spotted alfalfa aphid.

**Placoid sensilla**	**Preimmune serum (control)**	**Antisera**		
		**OBP6**	**OBP7**	**OBP8**
6th segment placoid	–	+++	++	+
5th segment placoid	–	–	–	–
3th segment placoid	–	++	+	+

*–, no significant labeling; +, 10–20 grains/μm^2^; ++, 20–30 grains/μm^2^; +++, more than 30 grains/μm^2^.*

**FIGURE 5:**
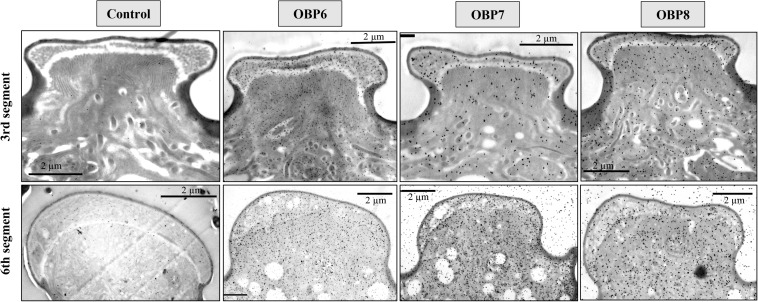
Immunocytochemical localization OBP6, OBP7, and OBP8 in sensilla placoidea on the 3rd and 6th segments of antennae in spotted alfalfa aphid. The preimmune serum was used as control. The antiserum against OBP6 of *T. trifolii* specifically and strongly labeled sensilla placoidea on the 6th segment and 3rd segment. The lymph of placoid sensilla on the 3rd segment was also labeled specifically by OBP8, but weaker than OBP6. The antiserum against OBP7 showed relatively weak, but significant staining specifically in the placoid sensilla of the 3rd antennal segment. Dilution of primary antibody was 1:1,000, and that of the secondary antibody was anti-rabbit IgG conjugated with 10 nm colloidal gold granules at a dilution of 1:20; the same below.

The results showed that the antiserum against OBP6 of *T. trifolii* specifically and strongly labeled sensilla placoidea on the 6th segment and 3rd segment. The gold granules are predominately distributed between the outer and inner cuticles, with a grain density of 35 and 28 grains/μm^2^ on the 6th segment and 3rd segment, respectively (Figure 5). The lymph of placoid sensilla on the 3rd segment was also labeled specifically by the antiserum against OBP8 (Figure 5), with a grain density of 17 grains/μm^2^. The antiserum against OBP7 showed relative weakness but significant staining specifically in the placoid sensilla of the 3rd antennal segment. Finally, very weak and barely significant labeling was observed with the antiserum against OBPs in the sensilla placoidea of the 5th segment (data were not shown).

**FIGURE 6:**
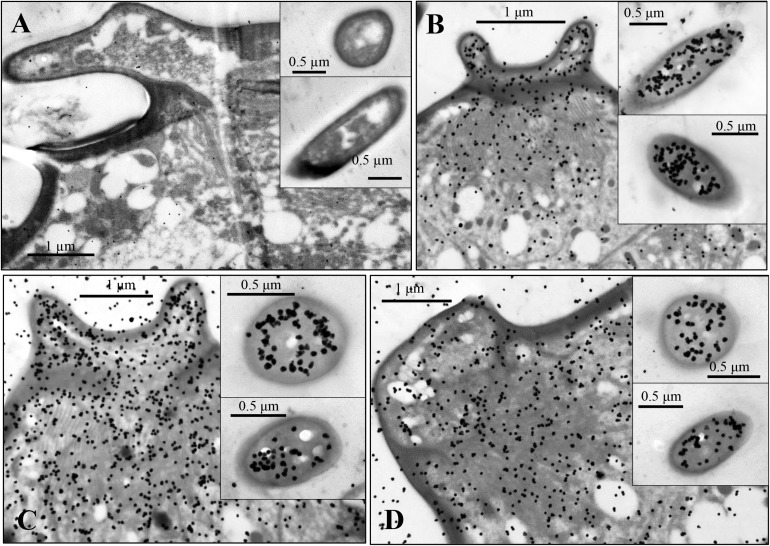
Immunocytochemical localization of OBP6 **(B)**, OBP7 **(C)**, and OBP8 **(D)** in sensilla stellate on the antennae of spotted alfalfa aphid. The preimmune serum was used as control **(A)**. OBP6, OBP7, and OBP8 are densely labeled in the sensillum lymph of stellate sensilla and appear to be colocalized in the same sensillum while preimmune serum shows no labeling at all. Dilution of the primary antibody was 1:1,000, and that of the secondary antibody was anti-rabbit IgG conjugated with 10 nm colloidal gold granules at a dilution of 1:20.

The stellate sensilla on the 6th segment were also labeled by OBP6, OBP7, and OBP8 antisera, mainly in the branches and sensillum lymph surrounding the dendrites (Figures 6).

## Discussion

In this study, we investigate the structure, morphology, and distribution of sensilla and the expression of three OBPs (OBP6, OBP7, and OBP8) in the antenna of SAA *T. trifolii*.

Similar to other aphids, the antennae of SAA *T. trifolii* also contained six segments. Based on external morphology, the antennae sensilla of SAA are classified into four types, including placoid sensilla, stellate sensilla, coeloconic sensilla, and trichoid sensilla. One striking observation is the presence of stellate sensilla on the 6th segment of primary rhinaria in this aphid. To our best knowledge, stellate sensilla were only identified in aphid species in the subfamily Drepanosiphinae (Shambaugh et al., 1978). Compared to other aphids, these aphids present two stellate sensilla but with two small placoid sensilla absent (Table 1), indicating these sensilla may be substitutes for the latter sensilla. The ultrastructure of these two type sensilla supported that they were similar, in that both of them are with three bipolar neurons (Ban et al., 2015). Similar to placoid sensilla, stellate sensilla also have many pores on the surface (Shambaugh et al., 1978; Ban et al., 2015), suggesting their chemosensory function (Sun et al., 2013). As previously described, we also observed the presence of multiple pores on the surface of the outer cuticle of placoid sensilla (Bromley et al., 1979), indicating they are typical olfactory chemoreceptors (Steinbrecht, 1984). We found that the number of secondary rhinaria (placoid sensilla) on the antenna are very similar between alate and apterous *T*. *trifolii* (Table 1), while secondary rhinaria were seldom found in apterous morphs of other aphids such as *M. persicae* and *A. pisum* (Table 1; Shambaugh et al., 1978; Sun et al., 2013; Ban et al., 2015; De Biasio et al., 2015). In addition, the ultrastructure of secondary rhinaria (placoid sensilla) is also very similar between alate and apterous morphs in *T. trifolii*, and further studies need to confirm this phenomenon in other aphids. Coeloconic sensilla have been reported that they are involved in thermo-/hygroreceptive functions in both Lepidoptera and Diptera (Sutcliffe, 1994). Similar to what was described in other aphid species (Bromley et al., 1980), two types of trichoid sensilla have been reported in our study, which could be involved in mechanosensing and/or in contact chemoreception (Bromley et al., 1980; Sun et al., 2013; Ban et al., 2015). Type II trichoid sensilla localized on the antennal tip and crowned by five blunt tipped uniporous hair, implying a gustatory function for these sensilla (De Biasio et al., 2015). The ultrastructure of coeloconic sensilla and trichoid sensilla needs to be more studied in the future.

Immunocytochemistry experiments have been used widely to study the location of OBPs in insects (Steinbrecht et al., 1995; Laue, 2000; Zhang et al., 2001, 2018; Zhu et al., 2016). Most literatures showed that OBP was usually found in sensilla, which have many pores on their surface (Steinbrecht et al., 1995; Laue, 2000; Zhang et al., 2001; Sun et al., 2013; Zhu et al., 2016). In herein, we selected three OBPs to investigate the expression pattern on the antennal sensilla. The results showed high expression of OBP6, OBP7, and OBP8 in the antennal placoid and stellate sensilla of adults, supporting a chemosensory role for these proteins in detecting alarm pheromones, plant volatiles, or sex pheromone (Bromley et al., 1979; Sun et al., 2013; De Biasio et al., 2015). The alarm pheromone is released by aphids in the presence of danger and induces other individuals to immediately leave the host plant. The alarm pheromone, (*E*)-β-farnesene has been found in all studied species of subfamilies Aphidinae and Chaitophorinae, while germacrene A was identified only within the genus Therioaphis of the subfamily Drepanosiphinae (Bowers et al., 1972, 1977; Nault and Bowers, 1974). Our results indicated that stellate sensilla might be involved in sensing alarm pheromone germacrene A. An early study suggests that OBP7, together with OBP3, is involved in the perception (*E*)-β-farnesene in both *M. persicae* and *A. pisum* (Sun et al., 2012; Zhang et al., 2017b). Our immunocytochemical results showed that placoid and stellate sensilla are strongly labeled by antibodies against OBP6 and significantly labeled by those against OBP7 and OBP8, suggesting that OBP6, OBP7, and OBP8 may sense alarm pheromone germacrene A. OBP3 has been reported high-binding affinity to the (*E*)-β-farnesene which is the only component of the alarm pheromone in *M. persicae* and *A. pisum* (Qiao et al., 2009; Sun et al., 2012). Whether OBP3 is involved in germacrene A perceiving is still unclear, which needs further work to decipher it. Further studies of molecular biology are also necessary to clarify the function of stellate sensilla in the alfalfa spotted aphid.

Overall, we identified the main sensilla types on the antenna of alfalfa spotted aphid, whose alarm pheromone is different from other aphids. Using TEM, we clarified the ultrastructure of stellate sensilla, which is absent in other aphid besides the subfamily Drepanosiphinae. Our findings will enrich recognition to the antennae sensilla of aphids. In addition, our study will provide insights into determining new strategies for control of these worldwide pests by interfering with their chemical communication.

## Data Availability Statement

The original contributions presented in the study are included in the article/Supplementary Material, further inquiries can be directed to the corresponding author/s.

## Author Contributions

LB conceived the project and designed the research. LS, YL, and YS performed the research. LS, XW, and LB analyzed the data and wrote the manuscript. All authors reviewed and approved the manuscript for publication.

## Conflict of Interest

The authors declare that the research was conducted in the absence of any commercial or financial relationships that could be construed as a potential conflict of interest.

## Supplementary Material

Supplementary Figure 1 The abundance and distribution of different sensilla on the antenna of spotted alfalfa aphid.
