# Supplementary figures and images for: Characterization of Antennal Sensilla and Immunolocalization of Odorant-Binding Proteins on Spotted Alfalfa Aphid, Therioaphis trifolii (Monell)
Source: Front Physiol. 2020 Dec 17;11:606575. doi: 10.3389/fphys.2020.606575 (PMC7773607; doi:10.3389/fphys.2020.606575)

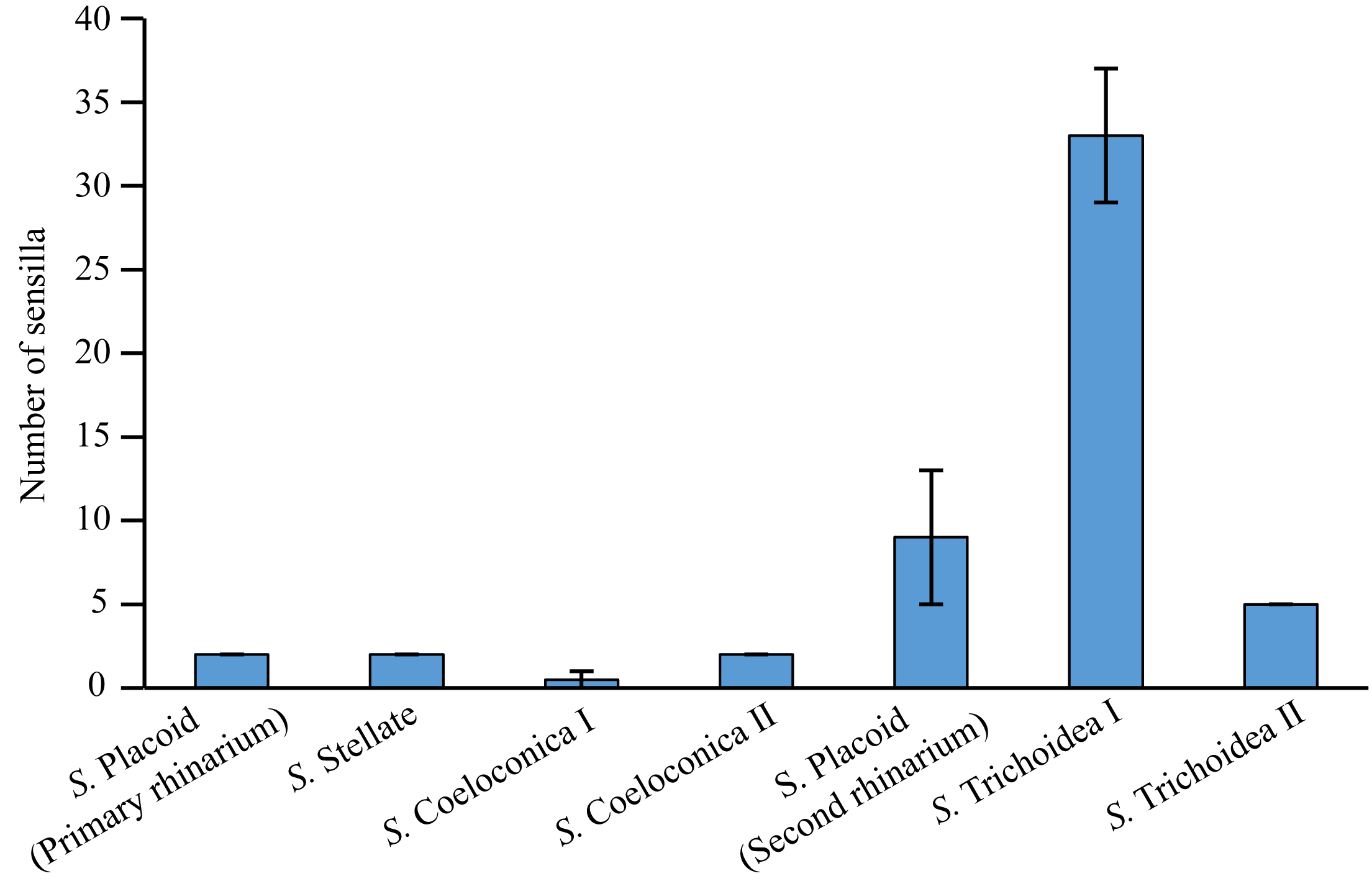

Supplement: Supplementary Figure 1 — The abundance and distribution of different sensilla on the antenna of spotted alfalfa aphid. [file Image_1.TIF]
